# Hospital Meetings, &C.

**Published:** 1898-04-09

**Authors:** 


### HOSPITAL MEETINGS, &C.

### ROYAL ORTHOPÆDIC HOSPITAL.

Sir Walter Gilbey, Bart., presided at the fifty-ninth annual meeting of the governors of this institution, held at the hospital on Wednesday, March 30th. The financial report is not of a satisfactory character, the accounts showing an excess of expenditure over income amounting to £285. The committee state that unless increased support is soon forthcoming it will be impossible to keep the whole of the 50 beds open, although the number of applicants for admission increases year by year, and on December 31st there were 43 names on the rotation list awaiting admission, the largest number yet recorded. The average annual deficiency during the past five years has been £280, and with regard to 1897 the report states that when H.R H. the Prince of Wales issued his appeal it was feared by many that the new scheme would divert much direct support from the hospitals, though at the same time it was expected that any loss would be made up by a liberal award from the fund. This hospital, however, has been awarded £61 5s., and the committee add that "as the charitable portion of the income, which was £911 in 1896, has fallen to £768, the award has not made up the deficiency." The total charges for the year are lower than in any year since the institution maintained the present number of beds. During 1897 170 in-patients were admitted, as against 153 in 1896. In the out-patient department there were 721 new cases, as against 706, and the attendances numbered 3,443, compared with 3,190. While the average period of treatment in the wards has been reduced from 104 to 101 days, the average number of beds occupied has increased from 44 to 47. The report was unanimously adopted.

### LONDON THROAT HOSPITAL.

Mr. John Alexander presided at the eleventh annual meeting of this institution, held at the hospital on Wednesday, March 30th. According to the report, the ordinary receipts, including £779 received from patients, amounted to £1,197, and the payments to £1,170. The actual expenditure, taking into consideration outstanding accounts, was £1,240. On account of the special fund for furnishing and opening the new wards £796 was received. In May, the committee state, the new wards were open, after being closed for some time for sanitary alterations and enlargement, and during the subsequent eight months 306 in-patients were treated. The new out-patients numbered 2,594, and the total attendances 13,132. In referring to the report, the Chairman said it might be asked, How does this hospital exist? The answer was that it existed by the work gratuitously performed by the medical staff, and by the financial support accorded to it in the shape of donations and subscriptions. He appealed to those present to remember the London Throat Hospital when making up their charitable list. Dr. Woakes, dealing with the history of the institution, said its founders had been connected with the hospital in Golden Square, and when so many members of the staff of the Golden Square Hospital seceded it was deemed desirable that they should not disperse. Eventually they found in this hospital a new centre for work which had been carried on ever since. Dr. Woakes further remarked that they had had a number of secretaries, and had suffered through some of them, but he was happy to say the lady now holding the post carried out the duties admirably. A letter from Mr. W. Burdett-Coutts, M.P., resigning the chairmanship of the General Committee on the ground of pressure of engagements, was read, and the meeting closed with a vote of thanks to the chairman.

### SAMARITAN FREE HOSPITAL.

The fifty-first annual report of the committee was presented at the meeting held at the hospital on Tuesday, March 29th. Dr. C. H. F. Rorth occupied the chair. The income for the year, excluding a loan of £700 from the bankers, amounted to £7,030; and the expenditure, minus the repayment of an overdraft of £803, to £6,751. Included in the receipts is a sum of £824, collected in amounts of £1 each by former patients anxious to render assistance to the hospital. The number of in-patients received was 542, 315 being medical and 227 surgical cases. Both on the medical side and on the surgical side some very difficult cases were treated, necessitating a prolonged stay in the hospital. There were in all 3,433 out-patients, and the total number of attendances was 22,178. In referring to the work of the institution, the report remarks that several cases of disease of unusual rarity as well as severity have been under treatment in the hospital during the past year. The operations may be witnessed upon application by qualified medical visitors, provided they sign a declaration that they are free from septic influences. A large number of British and foreign surgeons avail themselves of this privilege. It is further pointed out, with regard to the in-patient department, that it will soon be necessary to consider what means may be adopted to supply the increasing demand on the accommodation. The most pressing need of the hospital, however, is a new out-patients' department. About one hundred patients are seen every day by the medical officers, but the work, the committee remark, is seriously interfered with owing to the want of room; and if better accommodation cannot be obtained it will be necessary to limit the number of cases. The committee, therefore, emphasise the necessity of raising funds for the building of a new department which will be worthy of the institution.

### METROPOLITAN HOSPITAL.

The report of the committee presented to the governors at the meeting held on Tuesday, the 29th ult., under the presidency of Mr. Charles J. Thomas, states that 1897 was not so prosperous a year for the hospital as had been anticipated. The deficiency on the year's working was about £1,500, and the liabilities at December 30th amounted to £11,176, as against £9,523. The committee remark that in view of the large amount raised for the Indian Famine Fund and the Prince of Wales's Fund it is perhaps only natural that the hospital should for the time being have lost a certain amount of support. A strong effort will be made at the festival dinner on April 18th, at which the Lord Mayor has consented to preside, to raise funds to pay off the heavy debt and also to enable the committee to open additional beds. Touching on this point, Mr. Goodsall observed that if the income could be increased 50 per cent. it would be possible to double the number of beds in use, since there were certain charges that the opening of additional beds would not increase. There has been a further expansion in the work of the hospital during the year, the in-patients numbering 1,055, as against 1,013. The out-patients numbered 27,807, and the total attendances 100,793. Included in these figures are 164 Jewish in-patients and 8,957 attendances on Jewish out-patients. In this connection it may be said that the hospital has received a donation of £1,000 from the executors of the late Mr. Barnato. The committee are now inquiring into the working of the Provident Department, which was established some ten years ago, the object being to ascertain whether its usefulness can be extended. At the end of 1897, the number of members belonging to this department was 4,585. The total attendances amounted to 28,074, and 7,302 visits were made at the homes of members, the net receipts from the department for the year being £181. On July 20th the convalescent home at Cranbrook was opened, Miss E. Stirling-Hamilton being appointed as matron, and Miss Mabel Cave has been appointed matron of the hospital itself in place of Miss Ellen B. Kingsford, whose resignation the committee received with regret. The nursing staff has also been increased, so that each nurse may have three hours off duty every day, one half-day a week, and one whole day a month. The night nurses have two days a month. Mr. Ernest Flower, M.P., seconded the adoption of the report, which was carried unanimously.

### WEST-END HOSPITAL FOR DISEASES OF THE NERVOUS SYSTEM.

The annual general meeting of this institution was held at the hospital on Friday, March 25th, under the presidency of Major-General Mercer. According to the committee's report, the total ordinary income for the year amounted to £2,451, and the ordinary expenditure to £2,864. The extraordinary receipts consisted of £550, repaid to the general fund by the building fund; and the extraordinary expenditure amounted to £192. Notwithstanding the loss of many subscribers during the year, there is an increase in the total number and in the amount subscribed. On the other hand, donations fell from £851 in 1896 to £637 in 1897, and the committee remark that this is doubtless attributable to the extraordinary demand made upon the public by the Prince of Wales's Fund. From the Prince's Fund the hospital received a special donation of £96. The sum of £5,678 is due by the building to the general fund, which compares with £6,036 a year ago. In 1897 the in-patients numbered 296, an increase of 24, although the hospital was closed for 15 days, in consequence of an outbreak of chicken-pox among the children. There were 3,021 new out-patients, the number of massage treatments being 6,308 and of electrical treatments 9,467. The committee refer with satisfaction to the success of a series of post-graduate lectures, with practical demonstrations, held at the hospital during the year. After the ordinary business had been disposed of, resolutions were adopted making alterations in the rules so as to give governors for each guinea subscribed two letters entitling patients to two months' treatment. Formerly it was the practice to issue for each guinea three letters entitling the holder of each to a month's treatment, but as many patients suffering from nervous diseases require more than a month's attention the alteration referred to was decided upon.

### NORTH-WEST LONDON HOSPITAL.

The annual general meeting of this institution was held at the hospital on Monday, 4th inst., under the presidency of Mr. George Herring. The donations for the past year show a heavy falling-off as compared with 1896—£1,745, against £5,370—the annual subscriptions having meanwhile decreased from £740 to £725. The total income from all sources amounted to £3,581, and the expenditure to £4,064. Special attention was directed by the secretary to the fact that the administration expenses amounted only to £272, or less than 7 per cent. of the entire expenditure. Mr. Herring, in referring to the falling-off in the donations, observed that while heavy demands had been made on the public by the Jubilee and the Prince of Wales's Fund, the amount received from the Fund by the North-West London Hospital was only £350. Indeed, he thought that it would have been better for the hospital if there had been no Prince of Wales's Fund; but, of course, that was a matter of opinion. The Chairman also mentioned with satisfaction the low proportion of the cost of management, which, he said, would compare favourably with that of any other institution, and had brought them increased sums from the Hospital Sunday and Saturday Funds. Owing to the decrease in the income, it is proposed to hold an International Fancy Fair, under the patronage of the Princess Christian, at the Athenæum, Camden Road, on May 9th, 10th, and 11th. Stalls will be provided by the congregations of the churches and chapels in the north-west district, and there will also be a hospital stall, for which contributions are solicited. The work of the hospital during the year has been carried on without break or interruption, the in-patients treated numbering 560, and the attendances in the out-patient department 35,023, of which 17,276 were new cases. After the report had been adopted, a resolution was passed to the effect that every assistant physician must be a graduate in medicine of a university of the United Kingdom and a member of the Royal College of Physicians of London, not practising midwifery or pharmacy. In the original rules the word "or" stood in place of "and." A cordial vote of thanks to the medical and general staff brought the proceedings to a close.

